# ECRG2, a novel transcriptional target of p53, modulates cancer cell sensitivity to DNA damage

**DOI:** 10.1038/s41419-020-2728-1

**Published:** 2020-07-17

**Authors:** Harsh Patel, M. Saeed Sheikh, Ying Huang

**Affiliations:** Department of Pharmacology, State University of New York Upstate Medical University, 750 East Adams Street, Syracuse, NY 13210 USA

**Keywords:** Tumour-suppressor proteins, Apoptosis

## Abstract

Esophageal Cancer-Related Gene 2 (ECRG2) is a recently identified tumor suppressor, its regulation and involvement in DNA damage response are unknown. Here, we show that DNA damage-induced ECRG2 upregulation coincided with p53 activation and occurred in a p53-dependent manner. We identified two p53-binding sites within *ECRG2* promoter and found the promoter activity, mRNA, and protein expression to be regulated by p53. We show that DNA damage significantly enhanced p53 binding to *ECRG2* promoter at the anticipated p53-binding sites. We identified a novel natural *ECRG2* promoter variant harboring a small deletion that exists in the genomes of ~38.5% of world population and showed this variant to be defective in responding to p53 and DNA-damage. ECRG2 overexpression induced cancer cell death; *ECRG2* gene disruption enhanced cell survival following anticancer drug treatments even when p53 was induced. We showed that lower expression of *ECRG2* in multiple human malignancies correlated with reduced disease-free survival in patients. Collectively, our novel findings indicate that ECRG2 is an important target of p53 during DNA damage-induced response and plays a critical role in influencing cancer cell sensitivity to DNA damage-inducing cancer therapeutics.

## Introduction

Multiple extrinsic stress stimuli including environmental carcinogens and ultraviolet (UV) radiation and intrinsic factors such as metabolic reactive oxygen species (ROS) lead to DNA damage^1^. Upon detection of DNA damage, cells respond by activating complex signaling networks that results into either DNA repair and survival or cell death^2^. These signaling pathways play crucial role, not only in malignant transformation, but also in determining the therapeutic success of anticancer drugs^3^.

The tumor suppressor p53 serves as a central node of the cellular response to various stresses including DNA damage through transcriptional regulation of multiple downstream target genes^4,5^. Upon DNA damage, p53 protein is stabilized via post-translational modifications and binds to the response elements present within the promoters or introns of its target genes in a sequence-specific manner^6,7^. These genes are able to orchestrate an array of biological consequences such as cell cycle arrest (e.g., *p21*, *GADD45a*, *14-3-3σ*), autophagy (e.g., *DRAM*) and apoptosis (e.g., *BAX*, *PUMA*, *DR5*)^8^. Apoptosis induced by p53 activation is crucial for eliminating the cells with severe genomic aberrations and thereby preventing malignant transformation^8^. Moreover, apoptotic response elicited by p53 is critical for the effectiveness of chemo- and radiotherapy^5^. Thus, proper functioning of pro-apoptotic downstream targets is indispensable for the tumor-suppressive role of p53.

Several p53 target genes with pro-apoptotic activity have been identified over the past two decades. However, none of these genes was demonstrated to be the sole effector of p53-mediated cell death^8^. It was suggested that functional redundancy of multiple pro-apoptotic p53 targets may be necessary to achieve successful tumor suppression^9^. Conversely, it was proposed that distinct pro-apoptotic p53 target genes are activated in response to a variety of stress stimuli in a tissue/cell type-specific manner^5^. Therefore, discovery and characterization of newer pro-apoptotic targets will help refining the understanding of p53-mediated apoptotic program in response to various stress stimuli in the diverse cellular contexts.

Esophageal cancer-related gene 2 (ECRG2), or Serine Peptidase Inhibitor Kazal type 7 (SPINK7), is a member of SPINK family which is characterized by the presence of at least one Kazal-type serine peptidase inhibitor domain^10^. Human *ECRG2* is a part of the cluster comprising of seven *SPINK* genes located at chromosome 5q32, a target location of frequent chromosomal aberrations in various human malignancies^11,12^. Recent evidence indicates that ECRG2 functions as a tumor suppressor^13,14^. *ECRG2* expression was abundantly detected in normal human tissues including esophagus, oral mucosa, pancreas, stomach, colon, lung, and cervix^15^. However, the expression of *ECRG2* gene was significantly lower in multiple human cancers when compared to the corresponding normal tissues^10^. Genetic alterations (missense mutations, deletion/frameshift mutations) in the *ECRG2* gene were also reported in various human malignancies^13^. Previous studies have shown that ECRG2 suppresses migration, invasion, and metastasis of cancer cells via inhibition of urokinase-type plasminogen activator (uPA)/plasmin activity^16^. Cheng et al. reported that ECRG2 knockdown caused chromosomal instability and aneuploidy^17^. Moreover, co-administration of ECRG2 protein with cisplatin has been demonstrated to potentiate the anticancer activity of cisplatin in the esophageal cancer cells^18,19^. Our previous study has shown that overexpression of ECRG2 activates caspases and induces cancer cell death; ECRG2 promotes proteasome-mediated degradation of Hu-antigen R (HuR) oncoprotein, an mRNA-binding protein that is important for regulation of gene expression^13^. We also found that ECRG2 expression is strongly activated during DNA damage-induced cell death^13^. Currently, little is known about how ECRG2 is regulated to mediate its tumor-suppressive activity. The molecular basis of its role and regulation in DNA damage response is also unknown. In the present study, we have investigated these issues.

## Results

### ECRG2 mRNA and protein are induced by DNA damage

**Fig. 1:**
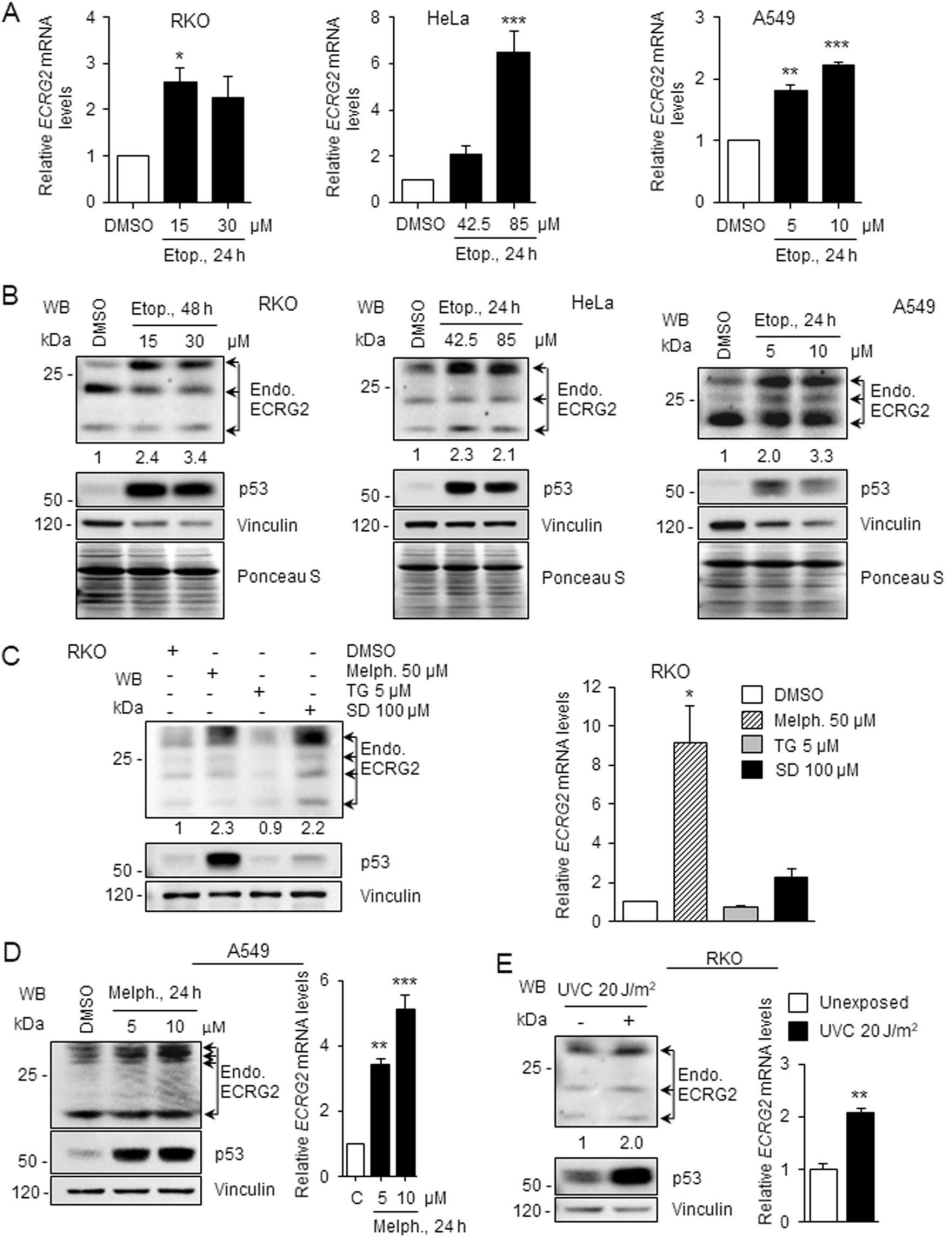
ECRG2 expression is induced by DNA damage. **a** *ECRG2* mRNA levels are induced by etoposide (Etop). *ECRG2* mRNA was analyzed by quantitative real-time PCR (qRT-PCR). **b** ECRG2 protein levels are induced by etoposide (Etop). Western blot (WB) analyses were performed using the antibodies specific for ECRG2 (upper), p53 (middle), and vinculin (lower). Numbers indicate fold induction in ECRG2 protein levels and were obtained by normalizing the relative band intensities of ECRG2 to that of vinculin (loading control). Ponceau S staining images corroborate total protein loading indicated by vinculin. **c**–**e** ECRG2 regulation by various stress including agents that induce or do not induce DNA damage. Melph melphalan. TG thapsigargin. SD sulindac sulfide. UVC ultraviolet C. qRT-PCR data in (**a**, **c**, **d**, **e**) are presented as mean ± SEM (*n* = 3). *p < 0.05, **p < 0.01, ***p < 0.001.

We have previously shown that ECRG2 overexpression induced apoptotic cell death and expression of a naturally occurring ECRG2-mutant (derived from patient tumor) promoted cancer cell survival following etoposide-induced DNA damage^13^. However, the molecular basis of ECRG2 regulation and its function in response to DNA damage remains to be elucidated. Figure 1a shows that *ECRG2* mRNA levels were significantly elevated in RKO, HeLa, and A549 human cancer cell lines by etoposide, a DNA-damaging anticancer agent^20^. Etoposide also upregulated ECRG2 at the protein levels in these cells (Fig. 1b). The cytotoxic effect of etoposide was also evaluated in these cell lines and the half-maximal inhibitory concentration (IC_50_) is presented in Supplementary Fig. S1. The specificity of ECRG2 antibody was demonstrated in our previous study^13^ and also is shown in Supplementary Fig. S2, which indicates that *ECRG2* knockdown by shRNA reduced the band-intensity of ECRG2 protein. In addition, p53 protein was also induced following etoposide treatment in the same cells (Fig. 1b). ECRG2 expression was also modestly upregulated by the treatments of UVC (20 J/m^2^) (Fig. 1e) and sulindac sulfide (SD)—a cyclooxygenase (COX) inhibitor, but not by thapsigargin (TG)—a Ca^2+^-ATPase inhibitor (Fig. 1c). In RKO cells (Fig. 1c), although SD (an NSAID) and melphalan (an alkylating agent that blocks DNA replication and induces DNA damage^21^) both induced ECRG2 protein level, SD only modestly enhanced *ECRG2* mRNA expression (~2 folds) with no p53 induction (Fig. 1c, left) whereas melphalan strongly induced *ECRG2* mRNA which was associated with strong induction of p53 (Fig. 1c, left). These results suggest that the mechanisms of ECRG2 induction by melphalan and SD may be different.

### *ECRG2* promoter is upregulated in response to DNA damage

**Fig. 2:**
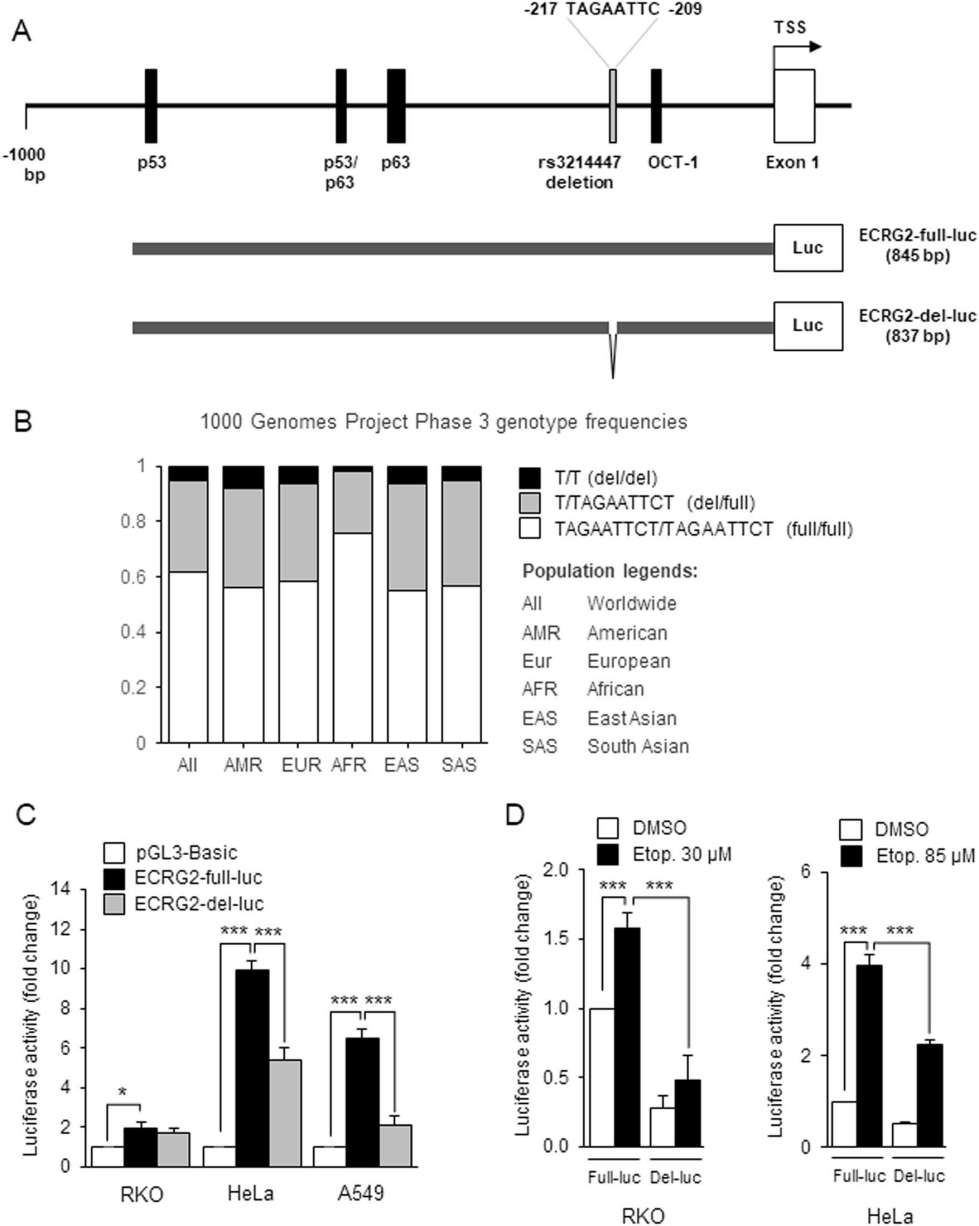
*ECRG2* promoter is induced by DNA damage. **a** Upper panel shows schematic illustration of *ECRG2* promoter and the potential binding sites (black bars) for transcription factors p53, p63, and OCT-1. A naturally occurring 8-bp deletion (gray bar) within *ECRG2* promoter is also indicated. TSS: transcriptional start site. Lower panel shows schematic representation of cloned promoter reporter constructs termed ECRG2-full-luc or ECRG2-del-luc (with missing 8-bp) are shown. **b** Genotype frequencies of *ECRG2*-promoter variants among the world and regional populations. Information is curated from the Ensembl website (www.ensembl.org). T/T means both alleles with deleted variant, T/TAGAATTCT means one allele with deleted variant, TAGAATTCT/TAGAATTCT means both alleles with full-length variant. (**c**) Basal promoter activity of the *ECRG2* promoter variants. Cells were transfected with luciferase constructs inserted without (pGL3 Basic) or with the *ECRG2* promoter sequences (ECRG2-full-luc, ECRG2-del-luc) for 24 h prior to the luciferase assay. **d** Etoposide treatment induces *ECRG2* promoter activity. Cells were transfected with indicated constructs for 24 h prior to the treatment with etoposide for additional 24-h. The data are presented as mean ± SEM (*n* = 3); *p ≤ 0.05, **p < 0.01, ***p < 0.001.

To examine the mechanism(s) involving *ECRG2* mRNA induction by DNA damage, we analyzed the *ECRG2* promoter (information retrieved from the human genome database^22^). Using the analytical tools from Genomatix^23^, JASPAR^24^, and PhysBinder^25^, we identified several potential regulatory elements within *ECRG2* promoter, which may play a role in *ECRG2* mRNA induction by DNA damage. For example, the upstream 1000 bp region of the *ECRG2* promoter (−1000 to +1) was predicted to harbor binding sites for p53, p63, and OCT-1 (Fig. 2a). *ECRG2* gene promoter has never been cloned and functionally characterized. Accordingly, we cloned *ECRG2* promoter using genomic DNA from A549 lung cancer cells and placed the promoter sequence corresponding to −845 to +1 upstream of the promoter-less luciferase reporter. Interestingly, following promoter cloning, we identified two naturally occurring variants of *ECRG2* promoter. Alignment of these variants revealed that the shorter variant (hereafter named as ECRG2-del) was missing eight nucleotides (TAGAATTC) at position −217 to −209 when compared with the longer variant (hereafter named as ECRG2-full) (Fig. 2a). Database analyses revealed that the variant sequence corresponding to the ECRG2-del exists in the database of Short Genetic Variations (dbSNP) designated as “rs3214447”^26^. Based on the information curated from 1000 Genomes Project Phase-3^27^, about 38.5% of world population harbor the “rs3214447” variant (8-nt deletion) in one or both alleles of the *ECRG2* promoter (Fig. 2b). Given that *ECRG2* promoter (either full-length or the deletion variant) has never been functionally characterized, next, we investigated how these two promoter variants are regulated. As shown in Fig. 2c, the luciferase activity of the ECRG2-full-luc (long variant) reporter construct was significantly higher than that of the promoter-less control construct pGL3-Basic (Fig. 2c). Furthermore, in HeLa and A549 cells, the activity of ECRG2-full-luc was markedly higher than that of ECRG2-del-luc, i.e., the activity of ECRG2-full-luc was ~9 and 6-folds higher than that of the pGL3-basic control, while ECRG2-del-luc was ~5 and 2 folds higher than that of the pGL3-basic control, respectively (Fig. 2c). In addition, in response to etoposide treatment, the activity of both *ECRG2* promoter variants was induced, however, the activation of ECRG2-full-luc was more robust than that of ECRG2-del-luc (Fig. 2d). Our results thus demonstrate, for the first time, that (1) *ECRG2* promoter is activated by DNA damage, and (2) TAGAATTC deletion within the *ECRG2* promoter appears to negatively impact the *ECRG2* promoter response to DNA damage.

### ECRG2 expression is induced by p53

**Fig. 3:**
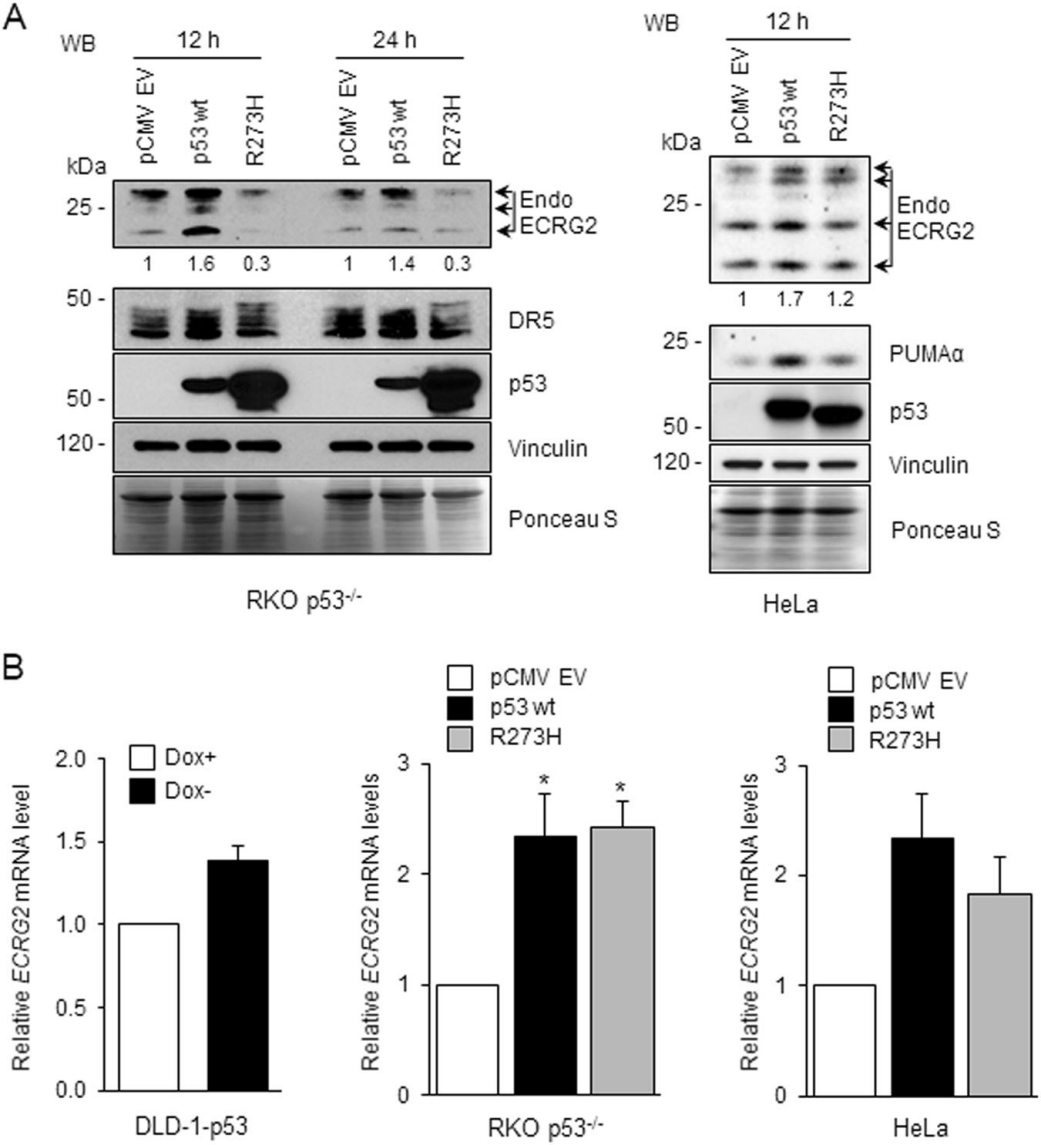
Overexpression of p53 induces ECRG2. **a** RKO p53^−/−^ and HeLa cells were transiently transfected with pCMV-emtpy vector, pCMV-p53-wildtype (wt), or pCMV-p53R273H mutant for the indicated duration and analyzed for expression of ECRG2 and other proteins by Western blotting. Numbers underneath the blots indicate fold induction in ECRG2 protein levels and were obtained by normalizing the relative band intensities of ECRG2 to that of vinculin (loading control). Ponceau S staining images corroborate total protein loading indicated by vinculin. **b** *ECRG2* mRNA analyses in cells expressing p53 wildtype and mutant constructs by qRT-PCR. RKO p53^−/−^ and HeLa cells were similarly transfected as described in **a**. p53-inducible DLD-1 (DLD-1-p53) cells were maintained in the doxycycline containing media (Dox+) prior to p53 induction. p53 induction was achieved by removal of doxycycline from the cell culture medium (3 h). Results of qRT-PCR are presented as mean ± SEM (*n* = 3); *p < 0.05.

As mentioned earlier, *ECRG2* promoter is predicted to harbor two p53-binding sites (Fig. 2a and Supplementary Fig. S3), thus, we investigated whether p53 regulates ECRG2. As shown in Fig. 3a (left panel), in RKO p53^−/−^ cells, ECRG2 protein level was significantly elevated following transfection with wild type (wt)-p53, but not with mutant-p53 (R273H)^28^. In HeLa cells, the extent of ECRG2 induction by wild type p53 overexpression was modest, but still, more than that caused by the mutant p53-R273H (Fig. 3a, right). The expressions of exogenous wild type-p53 and mutant- p53 are shown in Fig. 3a, and it appears that expression level of mutant-p53 (R273H) is higher in the RKO cells than that in HeLa cells. This could be due to the difference of cellular contents in these two different cell lines. In addition, the protein expression of PUMAα and death receptor 5 (DR5), two known p53-targets^29,30^, was also similarly regulated by wild type p53 or mutant p53-R273H in these cells (Fig. 3a). Figure 3b (left panel) shows that *ECRG2* mRNA was modestly elevated in p53-induced DLD-1 cells. *ECRG2* mRNA was also strongly induced in wild type p53-expressing RKO p53^−/−^ cells and HeLa cells (Fig. 3b, middle and right panels). Together, our results show that p53 positively regulates ECRG2 mRNA and protein expression.

### p53 directly binds to *ECRG2* promoter

**Fig. 4:**
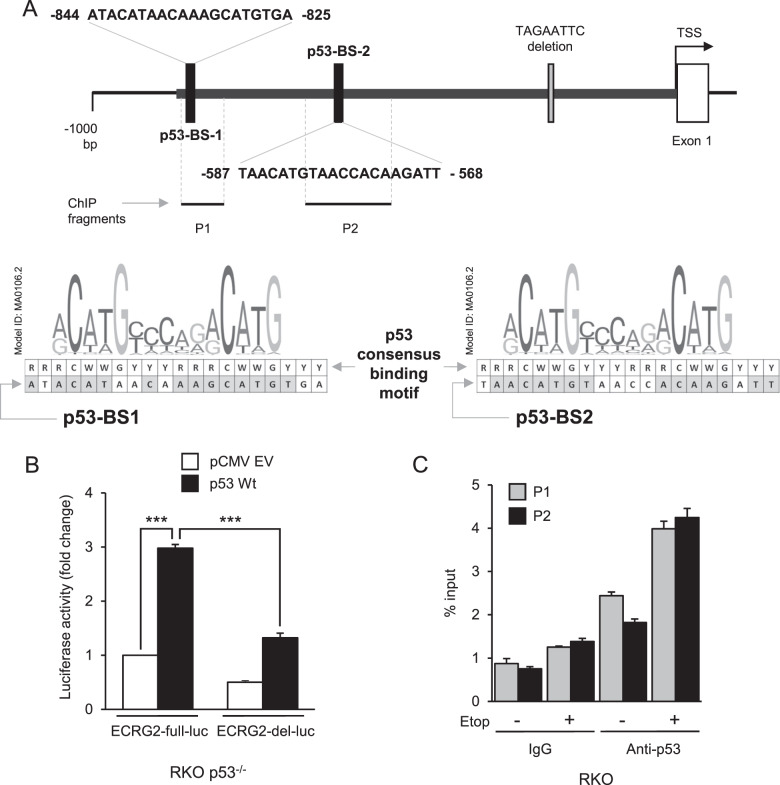
p53 directly binds to *ECRG2* promoter. **a** Schematic diagram of two putative p53-binding sites (p53-BS-1 and 2) within the *ECRG2* promoter. Alignment of p53-binding motif and two p53-BSs of *ECRG2* promoter are also indicated. For p53-binding motif, R = purine (A/G), WW = (A/T) or (T/A) and Y = pyrimidine^24,31^. The shaded boxes indicate the match between p53-binding consensus motif and p53-binding site sequence within *ECRG2* promoter. PCR amplified regions of the *ECRG2* promoter for the ChIP assays are also indicated. P1 flanking p53-BS-1; P2 flanking p53-BS-2. **b** p53 overexpression induces *ECRG2* promoter activity. RKO p53^−/−^ cells were transfected with empty vector (pCMV EV) or wildtype-p53 (p53 wt) together with ECRG2-full-luc or ECRG2-del-luc constructs for 24 h before harvesting for luciferase activity assays. Results are presented as mean ± SEM (*n* = 3). **c** p53 binds to P1 and P2 regions of *ECRG2* promoter as demonstrated by the ChIP assay. Cells treated with DMSO (vehicle) or etoposide (16 h) were fixed and harvested for genomic DNA extraction followed by chromatin shearing by sonication. Protein-bound-DNA fragments were immunoprecipitated by p53 antibody or control IgG. The immunoprecipitated DNA was analyzed by qPCR using primers flanking BS-1 or BS-2 regions (P1 and P2 respectively, shown in (**a**)). The data represents mean values of triplicate measurements (*n* = 2); ***p < 0.001.

As shown in Fig. 4a, *ECRG2* promoter harbors two putative p53-binding sites. The DNA sequence analysis revealed that the putative p53-binding site 1 (p53-BS1) and 2 (p53-BS2) within *ECRG2* promoter exhibit ~70% homology to the consensus p53 binding motif reported by El-Deiry et al.^31^, and even higher degree of homology was noted when BS-1 and -2 DNA sequences were aligned with Position Weight Matrix (PWM) of p53 defined by JASPAR database^24^. We thus examined whether p53 is capable of activating *ECRG2* promoter. As shown in Fig. 4b, exogenously expressed p53 in RKO p53^−/−^ cells significantly induced ECRG2-full-luc promoter activity by ~3 folds (*p* < 0.001). These results clearly demonstrate that p53 activates *ECRG2* promoter. The ECRG2-del-luc reporter with 8-nt deletion was also induced by p53, but the total output of the activity by ECRG2-del-luc was substantially lower (by ~50%) than that of ECRG2-full-luc. This could be due to the lower basal activity of ECRG2-del-luc, which shows that 8-nt deletion within *ECRG2* promoter affects its basal activity under the unstressed condition.

Next, we investigated whether p53 is capable of directly binding to the *ECRG2* promoter and to that end, used the chromatin immunoprecipitation (ChIP) assay. Using this assay, we sought to determine (1) whether p53 binds to *ECRG2* promoter at the BS-1 and BS-2 sites and (2) whether DNA damage increases p53 binding to the BS-1 and BS-2 sites. Figure 4c shows that p53 antibody precipitated p53-bound BS-1 and -2 specific DNA fragments (P1 and P2, respectively) under unstressed- as well as DNA damage-induced stress conditions. However, etoposide-induced DNA damage (Etop+) significantly increased p53 interaction with BS-1 and 2 within *ECRG2* promoter (Fig. 4c). Our results, for the first time, demonstrate that *ECRG2* gene is a direct transcriptional target of p53 and recruitment of p53 to *ECRG2* promoter is significantly enhanced under the DNA-damaging conditions. These results also indicate that ECRG2 is a part of the p53-mediated responses following DNA damage.

### p53 is important for ECRG2 induction in response to DNA damage

**Fig. 5:**
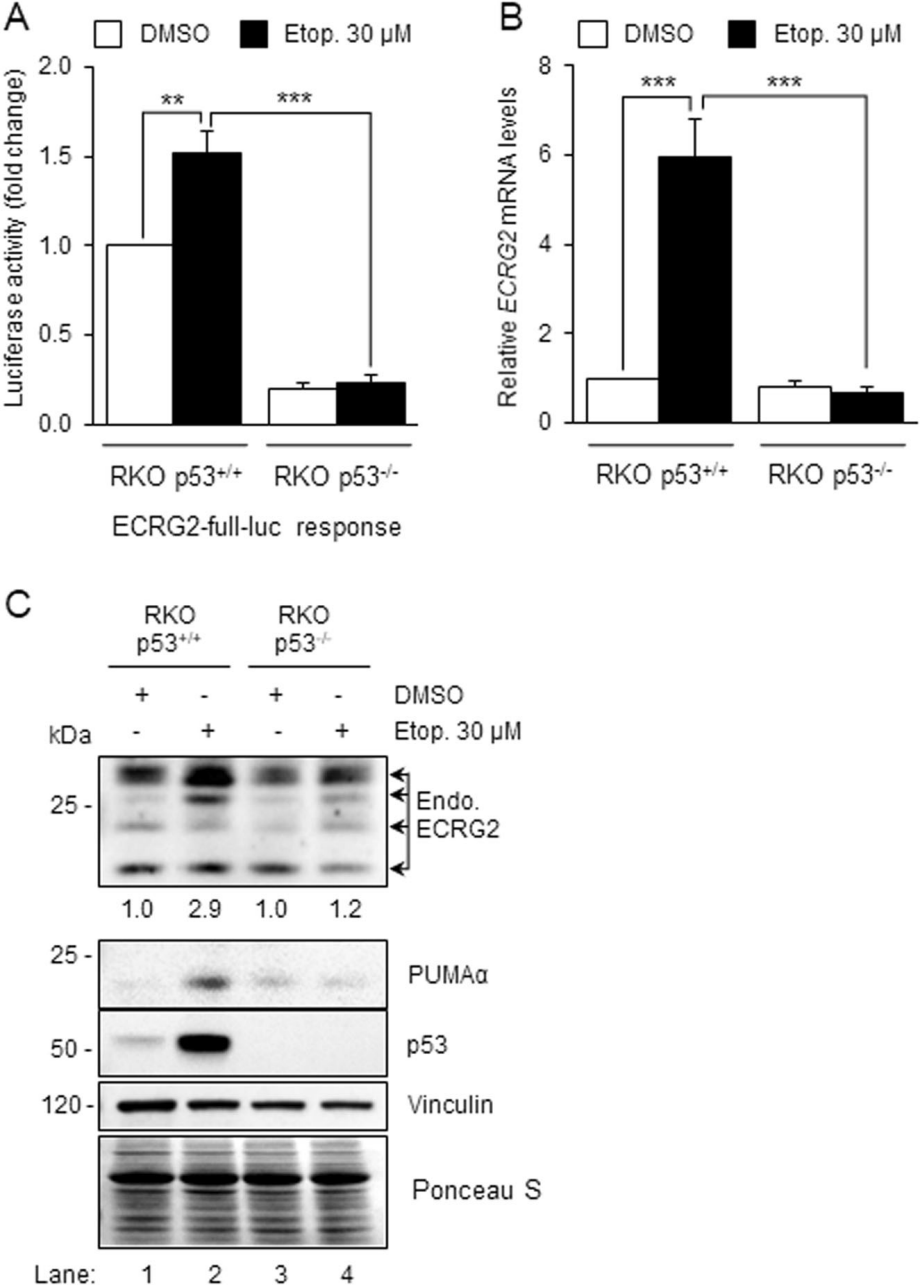
p53 is important for ECRG2 induction in response to DNA damage. **a** *ECRG2* promoter is activated by DNA damage in a p53-depenent manner. p53^+/+^ or p53^−/−^ RKO cells were transiently transfected with ECRG2-full-luc reporter plasmid for 24 hours. The cells were treated with 30 μM etoposide or DMSO for additional 24 h, and the promoter activity was measured by luciferase assay. The values are presented as mean ± SEM (*n* = 3). **b**, **c** Induction of *ECRG2* mRNA and protein in response to DNA damage is p53-dependent. p53^+/+^ or p53^−/−^ RKO cells were treated with 30 μM etoposide or DMSO for 24 hours. The mRNA levels were analyzed by qRT-PCR (**b**), and the protein levels were analyzed by Western blotting (**c**). Results in (**b**) represent mean ± SEM (*n* = 3). Numbers underneath the blot in (**c**) indicate fold induction in ECRG2 protein levels. Ponceau S staining was used to corroborate total protein loading indicated by vinculin. *p < 0.05, **p < 0.01.

We next investigated whether p53 is required for ECRG2 induction following DNA damage. As shown in Fig. 5a, *ECRG2* promoter (ECRG2-full-luc) activation induced by DNA damage (etoposide treatment, black bars) only occurred in RKO p53^+/+^ cells, but not in RKO p53^−/−^ cells. In addition, the basal activity of *ECRG2* promoter (ECRG2-full-luc) (white bars) was significantly lower in RKO p53^−/−^ cells than in RKO p53^+/+^ cells (Fig. 5a). ECRG2 mRNA and protein induction triggered by DNA damage (etoposide treatment) also occurred only in p53-proficient (RKO p53^+/+^) cells, but not in p53-deficient (RKO p53^−/−^) cells (Fig. 5b, c). Similarly, PUMAα (a p53 target) was also induced by etoposide treatment in RKO p53^+/+^ cells, but not in RKO p53^−/−^ cells. These results indicate that ECRG2 induction in response to DNA damage is p53-dependent.

### Effect of ECRG2 on cell growth and DNA damage-induced cell death

**Fig. 6:**
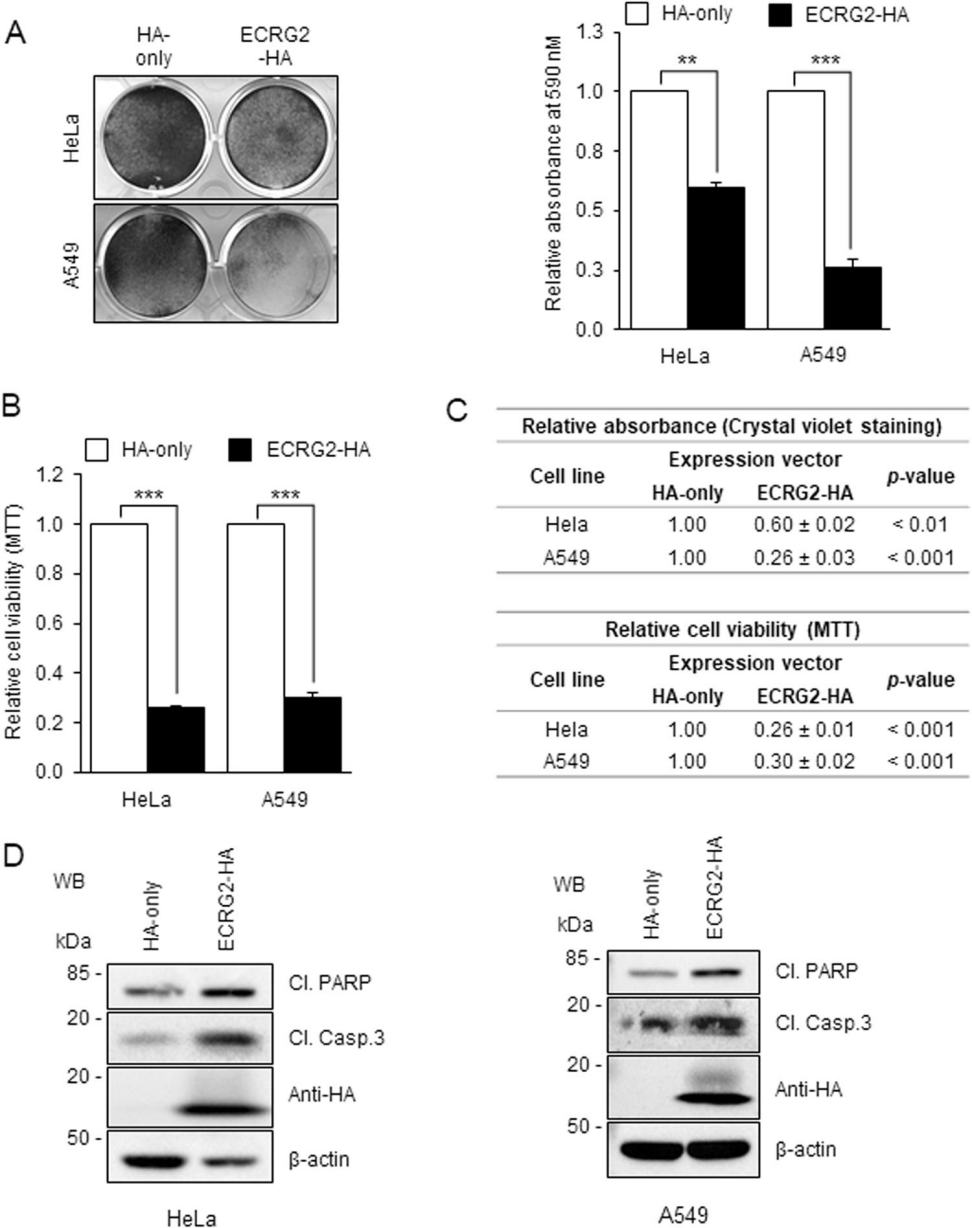
Overexpression of ECRG2 induces apoptosis, caspase 3 activation, and PARP cleavage. Relative cell viability of HeLa cells and A549 cells transiently expressing HA-only or ECRG2-HA for 48 h was analyzed through crystal violet staining (**a**) and MTT assay (**b**). Crystal violet staining images shown in (**a**) (left panel) were quantified by dissolving the stain and measuring the absorbance; the values of relative absorbance are plotted on the right. (**c**) Quantification data of relative absorbance from crystal violet staining experiment in (**a**) and relative cell viability analyzed by MTT in (**b**). The *p* values showing the statistical significance were calculated using two-tailed Student’s *t* test. The data in (**a**–**c**) are presented as mean ± SEM (*n* = 3). **d** HeLa and A549 cells transiently expressing HA-only or ECRG2-HA for 48 h were harvested for the protein analysis by Western blotting using the indicated antibodies. Cl. PARP: cleaved PARP; Cl. Casp.3: cleaved caspase 3; **p < 0.01, ***p < 0.001.

**Fig. 7:**
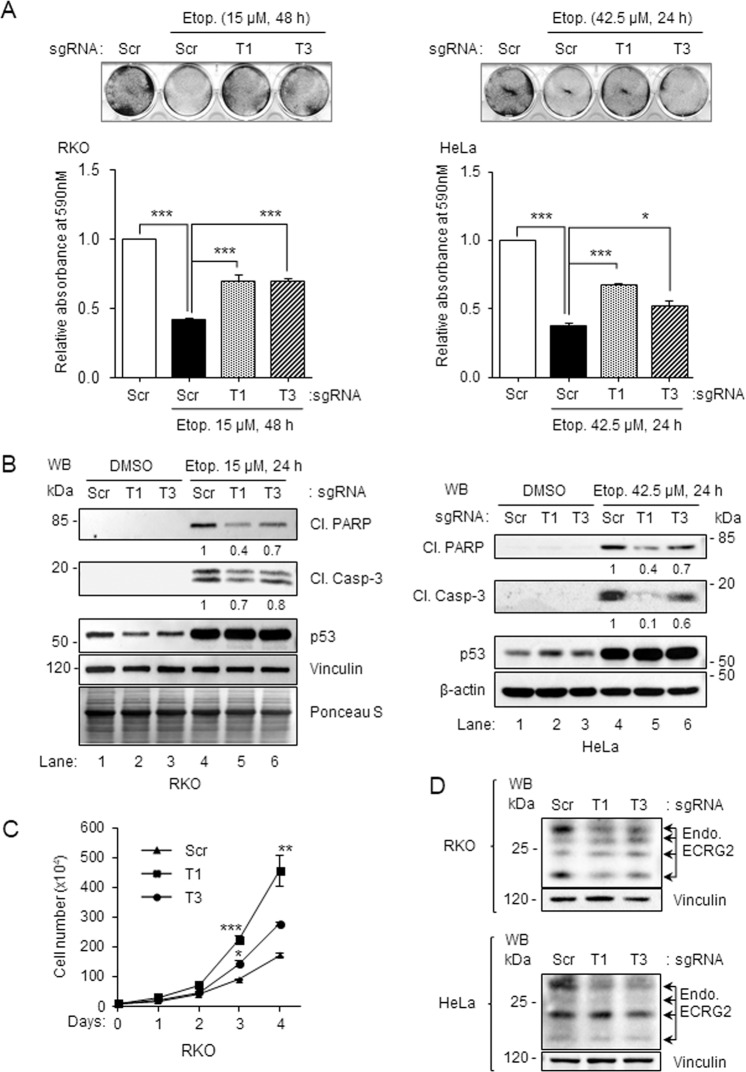
Loss of ECRG2 promotes cell survival following DNA damage. **a**, **b** RKO and HeLa cells were infected with lentivirus expressing scrambled sgRNA/Cas9 (control) or *ECRG2* gene-specific T1- or T3 sgRNA/Cas9 followed by selection with puromycin for at least 5 days. Equal numbers of control or *ECRG2*-gene-targeted cells (whole-cell populations) were seeded for 24 h followed by treatment with or without etoposide for additional 24 hours. In (**a**), cell viability was determined by imaging of crystal violet stained cells (top panel) and measuring the absorbance of dissolved stain (bottom panel). Values of relative absorbance represent mean ± SEM (*n* = 3). In (**b**), Western blot analyses were performed using the indicated antibodies. Cl. PARP: cleaved PARP; Cl. Casp-3: cleaved caspase-3. **c** Loss of ECRG2 expression promotes cell proliferation in RKO cells. Equal numbers of scrambled sgRNA/Cas9 cells or *ECRG2*-gene targeted cells (as described in (**a**)) were seeded and cultured for indicated days before evaluating for the rate of cell proliferation by counting live cells (trypan-blue exclusion assay). Each data point represents mean ± SEM of triplicate counts from a representative experiment. Additional experiments generated similar results (*n* = 2). **d** WB images showing the loss of ECRG2 expression in T1- or T3 sgRNA/Cas9 expressing cells. *p < 0.05, **p < 0.01, ***p < 0.001.

Figure 6a and b show that expression of exogenous ECRG2 induced strong growth suppression in both A549 and HeLa cancer cells. Overexpression of ECRG2 also triggered activation of caspase-3 and cleavage of PARP (Fig. 6d), which are indications of apoptosis^32^. We further investigated how ECRG2 may affect cell survival in response to DNA damage. CRISPR/Cas9-mediated gene disruption approach was utilized to target the *ECRG2* gene. The efficiency of *ECRG2* gene editing was evaluated by Western blotting (WB) (Fig. 7d) and by the commonly used mismatch cleavage assay^33^ (Supplementary Fig. S4). Figure 7 shows that disruption of endogenous ECRG2 markedly enhanced the survival of RKO and HeLa cells under etoposide-induced DNA damage (Fig. 7a) and also reduced the cleavage (activation) of caspase-3 and PARP (Fig. 7b). Under unstressed conditions, *ECRG2* gene disruption did not change the growth rate of *ECRG2*-targeted HeLa cells compared to the scrambled control cells (data not shown), but significantly accelerated the growth of *ECRG2*-targeted RKO cells (Fig. 7c). These results indicate that ECRG2 plays an important role in the regulation of cell survival in response to DNA damage and also affects the cell growth.

### Decreased *ECRG2* expression is associated with poor prognosis in human malignancies

**Fig. 8:**
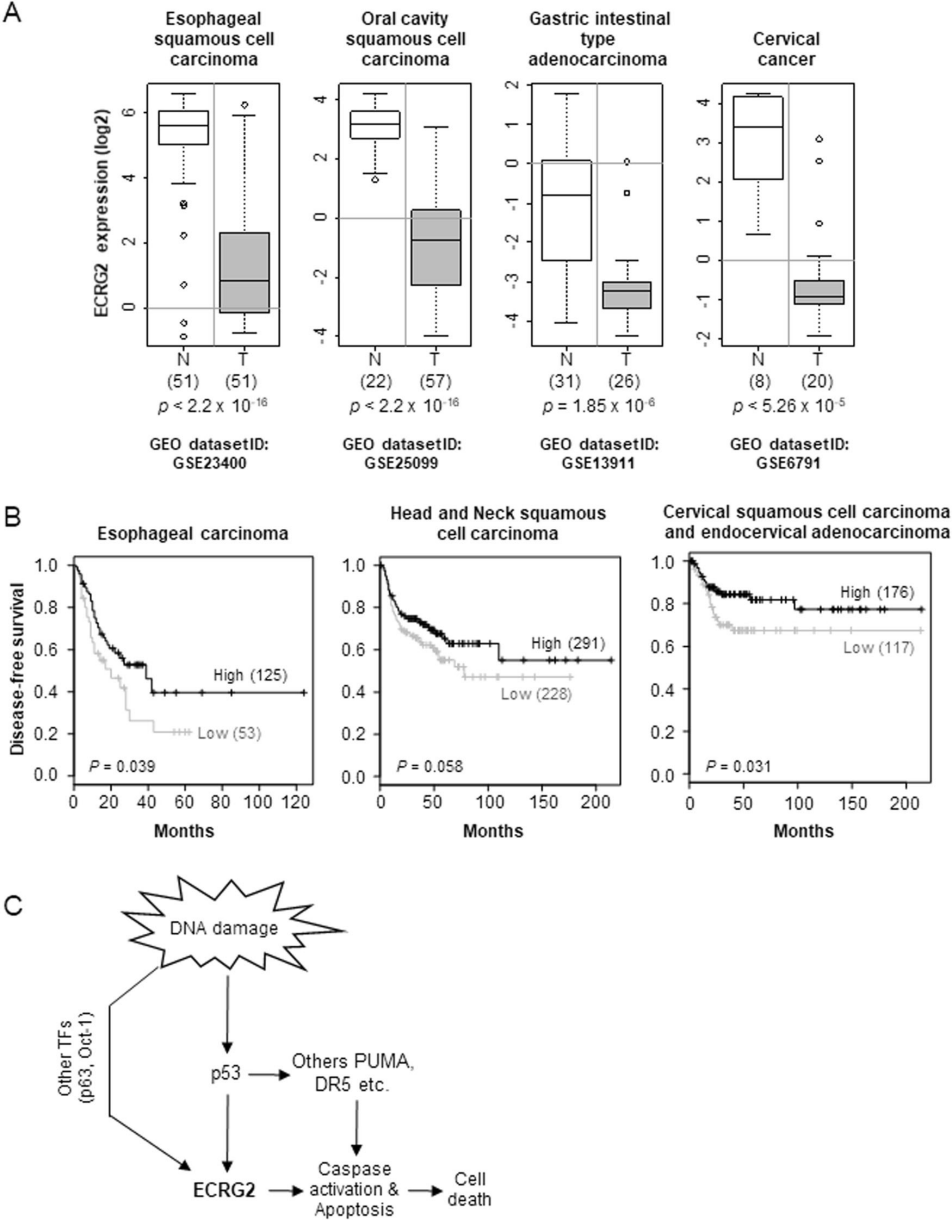
*ECRG2* expression is significantly decreased in human malignancies. **a** The results were plotted based on the information curated from the Oncomine online database^34^, which gathers the data from various previously published studies (see Materials and Methods). The boxplots display *ECRG2* mRNA expression in patient tumors (T) versus corresponding normal tissues (N). The *p* values were calculated using Two-tailed Student’s *t* test. **b** Low *ECRG2* expression is correlated with poor prognosis among the cancer patients. The patient information from the TCGA datasets of indicated cancer types was accessed and processed through GEPIA web-server^35^ (see “Materials and methods”). The cancer patients were segregated into two groups (high and low) based on *ECRG2* expression in the tumor samples. The Kaplan–Meier method was used to analyze and plot the rate of disease free survival between the two groups: solid black line for high, and grey line for low expression group. The patient count in each group is shown in the parentheses. The *p* values calculated using the log-rank test were generated by GEPIA web-server. **c** Proposed model of p53/ECRG2-mediated apoptosis following DNA damage as outlined in the “Discussion” section.

Using the information reported in the public databases, such as Oncomine^34^ and GEPIA-web server (based on TCGA datasets)^35^, we analyzed *ECRG2* expression status in human malignancies and its effect on prognosis among the cancer patients. Figure 8a shows that *ECRG2* expression was significantly lower in esophageal and oral squamous cell carcinoma, gastric adenocarcinoma, and cervical carcinoma when compared to corresponding normal tissues. Information obtained from the TCGA databases through GEPIA-web server indicated that the lower levels of *ECRG2* expression in several cancer types, i.e., esophageal cancer, head, and neck squamous cell cancer, as well as cervical squamous cell carcinoma and endocervical adenocarcinoma, was significantly correlated with reduced disease-free survival among the patients, whereas high levels of *ECRG2* expression coincided with better patient prognosis (Fig. 8b). These results suggest that the expression levels of *ECRG2* may not only influence tumor sensitivity to anticancer treatment, but also affects the prognosis of cancer patients.

## Discussion

In the present study, we have identified ECRG2 as a novel pro-apoptotic target of p53. We show that ECRG2 expression was upregulated by various agents (etoposide, melphalan, and UVC) that induce DNA damage and ECRG2 induction coincided with activation of p53 following DNA damage (Fig. 1). In addition, DNA damage-induced ECRG2 activation predominantly occurred only in p53-proficient cells, but not in p53-deficient cells (Fig. 5). DNA-damaging conditions promoted the recruitment of p53 to *ECRG2* promoter (Fig. 4), which then led to induction of ECRG2 mRNA and protein (Figs. 4 and 5). In this context, we also showed that elevated ECRG2 levels were coupled with activated caspases and induction of cell death (Fig. 6 and ref. ^13^). Taken together, our findings, for the first time, indicate that ECRG2 is instrumental in the regulation of apoptosis and serves as an integral component of p53-mediated cellular responses to DNA damage.

It is well-established that p53 serves as a central mediator for DNA-damage responses^36,37^. Following DNA damage, p53 is stabilized and activated by its phosphorylation and subsequently, transactivates target genes that mediate cell cycle control or apoptosis^37^. If the extent of DNA damage is beyond the repair capacity, cell death becomes imminent or the genetic errors are passed on to the daughter cells, accumulation of which can lead to cancer formation. Thus, p53 mediated cell death, via transactivation of apoptotic genes, is an important cellular event to ensure the genetic integrity of cells that averts cancer formation. In addition, induction of apoptotic genes by p53 is also critical for the effectiveness of anticancer agents that execute their effects via inducing DNA damage^5^. In this context, our current results indicate that ECRG2 plays an important role for the induction of cell death mediated by p53 following DNA damage. We have shown that disruption of ECRG2 significantly enhanced cell survival and prevented or reduced the cleavage (activation) of caspase 3 and PARP following etoposide treatment; such ECRG2-deficiency-associated changes occurred in wild type p53 cells (RKO), even when p53 was induced (Fig. 7a, b). These results demonstrate that ECRG2 is important for p53-mediated cell death in response to DNA damage.

A number of p53-targets genes such as *PUMA*^30^, *Noxa*^38^, *Bax*^39^, and *death receptor 5 (DR5)*^29^ are known to be important in p53-mediated apoptotic responses following DNA damage. Studies have shown that loss of PUMA and Noxa can render cancer cells resistant to DNA damage-induced apoptosis in cells with functional p53^40^. Wang et al. have also shown that silencing DR5 promoted resistance to 5-fluorouracil (5-FU) in tumor cells with wild type p53 (HCT 116)^41^. Although, they are all targets of p53 for the induction of apoptosis, the mechanisms involving regulation of cell death are various. For example, PUMA, Noxa, and Bax are BH-3 domain-containing proteins that regulate the intrinsic apoptotic signals and control cytochrome *c* release from mitochondria^42^. DR5, on the other hand, mediates the extrinsic pathway of apoptotic cell death^43^. In the case of ECRG2, our previous findings have shown that ECRG2 promotes ubiquitination and proteasome-mediated degradation of HuR protein^13^. HuR is an mRNA-binding protein; it regulates mRNA stability and protein translation of multiple genes that are important for cell growth and cell death^44^. Its targets include mRNAs of *XIAP*^45^, *Bcl-2*, and *Mcl-1*^46^, which are known to be important negative regulators of apoptotic signaling pathways. Studies have shown that HuR protein expression levels positively correlate with the expression of *XIAP*, *Bcl-2*, and *Mcl-1*; HuR knockdown reduces *Mcl-1* and *Bcl-2* expression and promotes cell death^45,46^. Further, higher expression of HuR has been associated with resistance to chemotherapy^47^. Thus, ECRG2-mediated HuR protein degradation could lead to enhanced cell death and this could be one of the important mechanisms by which ECRG2 executes p53-mediated responses following DNA damage.

In addition to p53, binding site predictions for p63 (a p53 homologue) and OCT-1 transcription factors were also found within *ECRG2* promoter region (Fig. 2a). Previous studies have shown that both p63^48^ and OCT-1^49^ are induced by DNA damage. Although, our current study was focused on investigating p53-mediated ECRG2 regulation, it is possible that p63 and/or OCT-1 may also contribute, at least in part, to upregulation of ECRG2 expression under DNA damage (Fig. 8c). Further studies are required to determine the exact role of p63 and OCT-1 in regulating ECRG2 expression under DNA damage.

Another novel finding from our current study indicates that *ECRG2* promoter allele with rs3214447 variant (TAGAATTC deletion) negatively impacts *ECRG2* promoter activity under unstressed condition as well as under DNA-damage (Fig. 2). Our discovery of rs3214447 variant in relation to its negative impact on ECRG2 promoter activity is highly significant. This finding is potentially important as information revealed by the 1000 Genomes Project Phase-3 indicates that about 38.5% of world population harbors one or both alleles with TAGAATTC deletion within *ECRG2* promoter (Fig. 2b). As shown in our studies, elevated ECRG2 expression induces cell death (Fig. 6 and Ref. ^13^); thus, the level of ECRG2 induction following DNA damage may determine whether cells commit apoptosis and also the extent of cell death. In this context, it will be interesting to investigate in the future whether cancer patients with the TAGAATTC deletion in the *ECRG2* promoter would exhibit strong apoptotic response following DNA damage-inducing anticancer drugs.

Our database analyses also revealed that *ECRG2* expression was significantly lower in multiple human malignancies compared to their corresponding normal tissues (Fig. 8a); and patients with lower expression of *ECRG2* in their cancers appear to exhibit reduced disease-free survival (Fig. 8b). Based on the evidence presented in our current study (Fig. 7), it is possible that lower *ECRG2* expression may confer survival advantage to cancer cells due to reduced drug sensitivity. Thus, cancer patients with deficiency in ECRG2 activation under drug treatments would be more likely to relapse. Along these lines, our previous findings had demonstrated that overexpression of a human cancer-derived ECRG2 mutant (V30E) not only failed to kill cancer cells, but also imparted resistance against multiple anticancer drugs^13^. Taken together, our present study highlights the important role of ECRG2 in p53-mediated apoptotic response as well as development of anticancer drug resistance in human malignancies.

## Materials and methods

### Antibodies, reagents, and treatments

The p53 (DO-1), PUMAα (B-6), and vinculin (7F9) antibodies were from Santa Cruz Biotechnology (Dallas, TX, USA). The antibodies against DR5 (D4E9), cleaved caspase 3 (D175) and cleaved PARP (Asp214) (D64E10) were from Cell Signaling Technology (Danvers, MA, USA). The HA tag (clone 3F10) and β-actin (clone AC-15) antibodies were from Roche Applied Science (Penzberg, Germany) and Sigma-Aldrich (St. Louis, MO, USA), respectively. ECRG2 antibody was generated in our laboratory as previously described^13^. The peroxidase-conjugated horse anti-mouse, goat anti-rat, and goat anti-rabbit antibodies were from Vector Laboratories (Burlingame, CA, USA). The cells were transfected using PolyJet or LipoJet reagents (SignaGen Laboratories, Rockville, MD, USA). The plasmid subcloning was performed using the restriction endonucleases from New England BioLabs (Ipswich, MA, USA). Etoposide, doxycycline, thapsigargin, and melphalan were from Sigma-Aldrich (St. Louis, MO, USA), and sulindac sulfide was provided by Merck (Rahway, NJ, USA). The cells growing in logarithmic phase were exposed to UVC as described previously^50^, except XL-1500 UV crosslinker (Spectronics, Westbury, NY, USA) was used as a source of UV radiation.

### Cells and culture conditions

The human cancer cell lines used in the study include RKO (colon), HeLa (cervix), and A549 (lung) and were obtained from NIH. RKO p53^−/−^ cells were kindly provided by Dr. Bert Vogelstein, Johns Hopkins School of Medicine, MD, USA. The cells were maintained in Dulbecco’s modified Eagle’s medium (DMEM) (Mediatech Inc., Manassas, VA, USA). The p53-inducible DLD-1 (DLD-1-p53) human colon cancer cells (kindly provided by Dr. Bert Vogelstein) were maintained in RPMI 1640 medium (Mediatech Inc., Manassas, VA, USA) with 40 ng/ml doxycycline prior to induction of p53. To induce p53 expression, the cells were washed 2-times with PBS, then incubated with RPMI 1640 medium without doxycycline. All culture media were supplemented with 10% fetal bovine serum (Gemini Bio-Products, West Sacramento, CA, USA), 1% penicillin-streptomycin solution (Mediatech), and 2 mM L-glutamine (Mediatech).

### Expression plasmids

pSRα-ECRG2-HA expression vector was generated in our lab as described previously^13^. The expression vectors pCMV-p53 wt, pCMV-p53 R273H, and pCMV empty vector were a kind gift from Dr. Bert Vogelstein.

### Quantitative RT-PCR

Two-step qRT-PCR assays were performed to analyze mRNA expression using the iScript cDNA Synthesis Kit and iQ SYBR Green Supermix from Bio-Rad (Hercules, CA, USA) as per manufacturer’s protocol. Ct values for *ECRG2* were normalized to the Ct values of *GAPDH* mRNA within the same sample, and fold changes in mRNA expression were determined by the ΔΔCt method as reported earlier^51^. The following primer sets were used: *ECRG2* forward: 5′-ATGAAGATCACTGGGGGTCTCCT-3′; reverse: 5′-TTAGCAACTTCCATCGTGAAGA-3′; and *GAPDH* forward: 5′-CACCATCTTCCAGGAGCGAG-3′; reverse: 5′-GCAGGAGGCATTGCTGAT-3′.

### Western blotting

Western blot analyses were performed as described previously^52^. Relative band intensities were determined by using Image Lab v4.1 software (Bio-Rad, Hercules, CA, USA), and fold changes are displayed under the relevant blot images.

### *ECRG2* promoter luciferase assays

*ECRG2* promoter-luciferase reporter vectors (ECRG2-full-luc and ECRG2-del-luc) were generated by PCR amplification of ~845 bp fragment upstream of *ECRG2* transcription start side (TSS) using the primer pair (5′-TCCATACATAACAAAGCATGTGATGGC-3′; 5′-ATCCCAGGTAAGGGGTCATG-3′) and human genomic DNA extracted from A549 human lung cancer cells as a template. The promoter fragments were then subcloned into pGL3-Basic vector (Promega, Madison, WI, USA) upstream of promoterless luciferase gene using KpnI and NheI enzymes. The promoter regulation analyses were performed using Luciferase Assay System (Promega, Madison, WI, USA) as described previously^53^.

### Chromatin immunoprecipitation (ChIP) assay

ChIP assays were performed using EpiTect ChIP OneDay Kit and Human p53 ChampionChIP Antibody Kit (Qiagen, Hilden, Germany). Cell fixation, lysis, chromatin shearing, antibody incubation and washing were performed as per manufacturer’s protocol. The antibody-bound chromatin fragments were isolated and purified as previously described^54^. Purified ChIP DNA was analyzed by qPCR and data were calculated and shown as %input as described earlier^55^. Following primers were used for qPCR:

p53 BS-1 forward 5′-AAAGCATGTGATGGCCACGAG-3′

p53 BS-1 reverse 5′-ATTAAAACTTCCAGCCCAGAGCA-3′

p53 BS-2 forward 5′-GCATGAACAGCTGACTACCAT-3′

p53 BS-2 reverse 5′-AAAAGGCTTGGTTATGTCGTGA-3′

### Cell viability and cell doubling time assay

The viability of cancer cells was determined by MTT assay or crystal violet staining. The MTT assays were performed as described previously^56^. Briefly, the cells were incubated with 0.5 mg/ml MTT for 45 min after the conclusion of the drug treatment of transient gene expression. The formazan crystals formed by viable cells were dissolved in the solubilization reagent [10% Triton-X 100 in acidic Isopropanol (0.1 N HCl)] and quantified by measuring the absorbance at 570 nm (background subtraction at 690 nm) using a microplate reader (Synergy H1 from BioTek, Winooski, VT, USA). In addition, the half-maximal inhibitory concentration (IC_50_) values were calculated for etoposide-treated cells using an online tool, Quest Graph™ IC_50_ Calculator^57^. The crystal violet staining was carried out using the protocol from Dr Ole Gjoerup’s lab^58^. Briefly, the cells were washed with PBS, fixed with 4% paraformaldehyde, and stained with 0.1% crystal violet staining solution. The excessive stain was washed off with water, and plates were air-dried overnight. The images were captured by scanning the plates using a flat-bed scanner (Epson Perfection V550). For quantification, 1 ml of 10% acetic acid solution was added to each well, and incubated 20 min with gentle shaking. The extracted stain was diluted 4x in water and absorbance at 590 nM was measured by the spectrophotometer (Bio-Rad, Hercules, CA, USA). For cell counting and trypan blue exclusion assay, equal number of cells from each group were seeded. The cells were harvested at the indicated time intervals, mixed with trypan blue (1:1) to identify and exclude dead cells and number of live cells were counted.

### CRISPR/Cas9 mediated gene disruption

*ECRG2* gene disruption was achieved using lentivirus-mediated CRISPR/Cas9 approach. Briefly, the cells were infected with lentivirus containing the *ECRG2* gene-specific (T1 or T3) guide RNA (gRNA) or scrambled-gRNA together with Cas9 nuclease. Puromycin selection was used to enrich the lentivirus-infected cell populations. All *ECRG2* gene-specific and non-specific (scrambled) constructs were obtained from Applied Biological Materials Inc. (Richmond, BC, Canada). The two different nucleotide sequence used to target human *ECRG2* in this study were: Target 1 (T1), 5′-AGTCAGAACCACAAACTGGT-3′ and Target 3 (T3), 5′-ATGAGGTACTCACAAGCTCT-3′. Lentivirus production and infection were performed as per Addgene protocols (www.addgene.org/protocols). Mismatch cleavage assay was performed using T7 Endonuclease I (T7E1) (New England Biolabs, Ipswich, MA, USA) to detect on-target insertion or deletion (InDel) events as per manufacturer’s instructions.

### Gene expression analyses of cancer patient datasets

Patient data from the Gene Expression Omnibus (GEO) datasets of following cancer types were accessed through the Oncomine online database (www.oncomine.org; accessed on August 16, 2019)^34^: (1) esophageal squamous cell carcinoma (ID: GSE23400), (2) oral cavity squamous cell carcinoma (ID: GSE25099), (3) gastric intestinal type adenocarcinoma (ID: GSE13911) and (4) cervical cancer (ID: GSE6791). Statistical significance of the difference between the mean expression values of patient tumors (T) and corresponding normal tissues (N) was calculated using the Two-tailed Student’s *t* test. The patient data from The Cancer Genome Atlas (TCGA) datasets of following cancer types were accessed and processed through the Gene Expression Profiling Interactive Analysis (GEPIA) web-server (http://gepia.cancer-pku.cn/; accessed on August 16, 2019)^35^: (1) esophageal carcinoma (ESCA), (2) head and neck squamous cell carcinoma (HNSC) and (3) cervical squamous cell carcinoma and endocervical adenocarcinoma (CESC). The patients were segregated into two groups (high and low) based on *ECRG2* expression in the tumor samples. The Kaplan–Meier method was used to analyze and plot the rate of disease free survival for both the groups. The long-rank tests were performed through the GEPIA web-server to compare the rate of disease free survival in both groups and estimate the statistical significance (*p* values).

### Statistical analysis

Statistical analyses were performed using the Rcmdr 2.5-3 package based on R software (version 3.6.1)^59^. Two-tailed Student’s *t* test or one-way ANOVA was used to compare the mean values. The values of *p* < 0.05 were considered to be statistically significant.

## Supplementary information

Supplementary figures legends

Supplementary Figure 1

Supplementary Figure 2

Supplementary Figure 3

Supplementary Figure 4
