# Supplementary material for: ECRG2, a novel transcriptional target of p53, modulates cancer cell sensitivity to DNA damage
Source: Cell Death Dis. 2020 Jul 17;11(7):543. doi: 10.1038/s41419-020-2728-1 (PMC7367829; doi:10.1038/s41419-020-2728-1)
Supplement: Supplementary file 1 — Supplementary figures legends [file 41419_2020_2728_MOESM1_ESM.docx]

**Supplementary Fig. S1**. Evaluation of etoposide-induced cytotoxicity. RKO, HeLa and A549 cells were treated with increasing concentrations of etoposide for 48 hrs. The cell viability was analyzed by MTT assay. (**a**) Dose-response curves were plotted and (**b**) IC_50_ values were estimated using the Quest Graph™ IC_50_ Calculator (https://www.aatbio.com/tools/ic50-calculator) based on the raw data obtained from MTT assay. Each data point in (**a**) is presented as mean ± S.D. (*n* = 3).

**Supplementary Fig. S2**. Western blot showing specificity of anti-ECRG2 polyclonal antibody. Endogenous ECRG2 in RKO p53^-/-^ cells was silenced by lentivirus mediated expression of small hairpin RNA (shRNA) targeting two different nucleotide sequences: KD-1, 5′-AACTGGTAGGTATGTGATGGG-3′ and KD-2, 5′-TCAGAACCACAAACTGGTAGG-3′ as previously described (Lucchesi *et al.*, PMID: 26434587). The cells were selected with puromycin for at least 5 days to enrich the population with ECRG2 knockdown.

**Supplementary Fig. S3**. Nucleotide sequence of human *ECRG2* promoter region. The potential binding sites for DNA-damage inducible transcription factors and the position of rs3214447 variant are underlined.

**Supplementary Fig. S4**. Detection of CRISPR/Cas9-mediated on-target insertion or deletion (InDel) events by mismatch cleavage assay. RKO and HeLa cells were infected with lentivirus expressing T1-, T3- or scramble sgRNA/Cas9. The CRISPR/Cas9 edited cell pool was enriched by selection with puromycin for at least 5 days. Untransfected cells (WT) or non-targeting (scramble) sgRNA were used as controls. Genomic PCR was performed to amplify the region flanking the CRISPR/Cas9 targeted sites (924 bp amplicon) using following primer pair: Forward: 5’-GAAGTGGCCAAGCAACATCTG-3’, and Reverse: 5’-ATGTCCGTGGTATTCTGGTCC-3’. The amplified PCR product was tested for CRISPR/Cas9 induced InDel mutations by T7 Endonuclease I (T7EI) assay. The cleavages at the heteroduplex mismatch sites were examined by agarose gel electrophoresis.
