# Supplementary figures and images for: ECRG2, a novel transcriptional target of p53, modulates cancer cell sensitivity to DNA damage
Source: Cell Death Dis. 2020 Jul 17;11(7):543. doi: 10.1038/s41419-020-2728-1 (PMC7367829; doi:10.1038/s41419-020-2728-1)

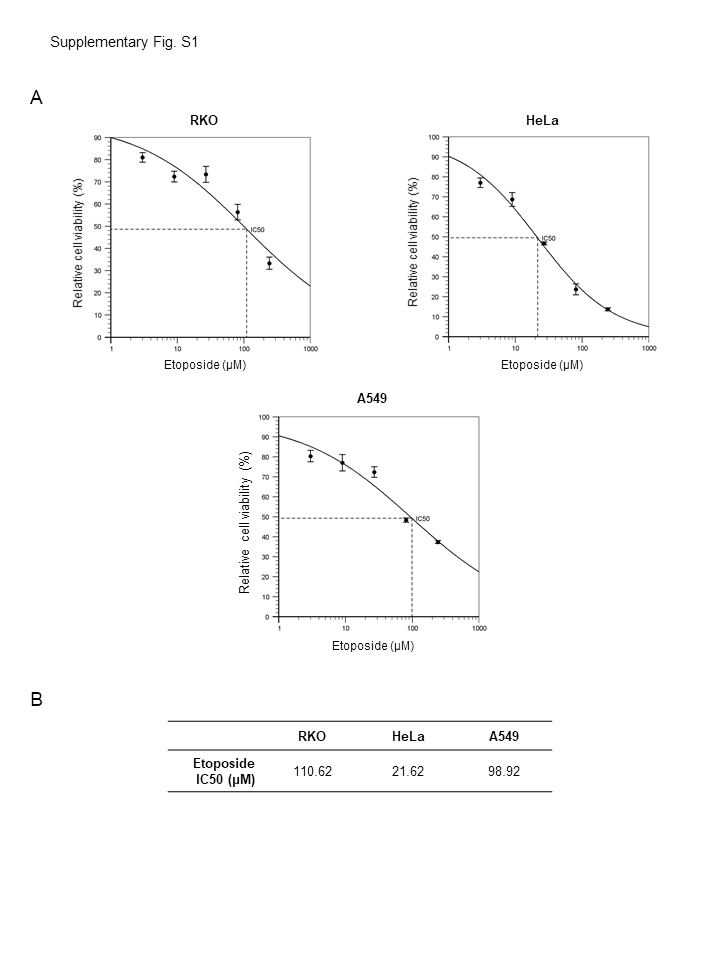

Supplement: Supplementary file 2 — Supplementary Figure 1 [file 41419_2020_2728_MOESM2_ESM.tif]

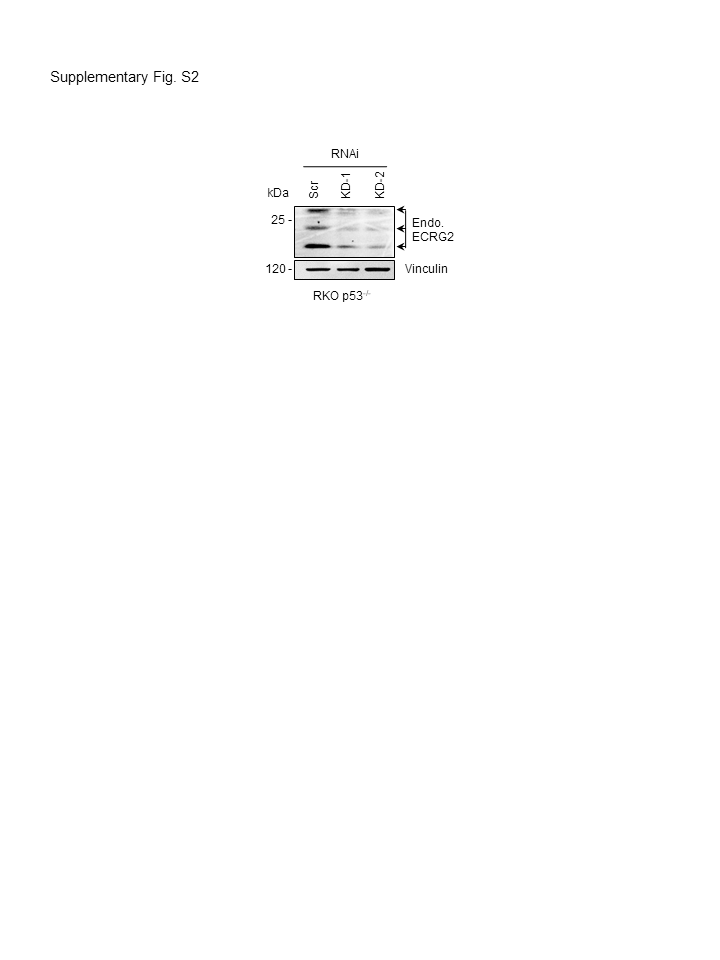

Supplement: Supplementary file 3 — Supplementary Figure 2 [file 41419_2020_2728_MOESM3_ESM.tif]

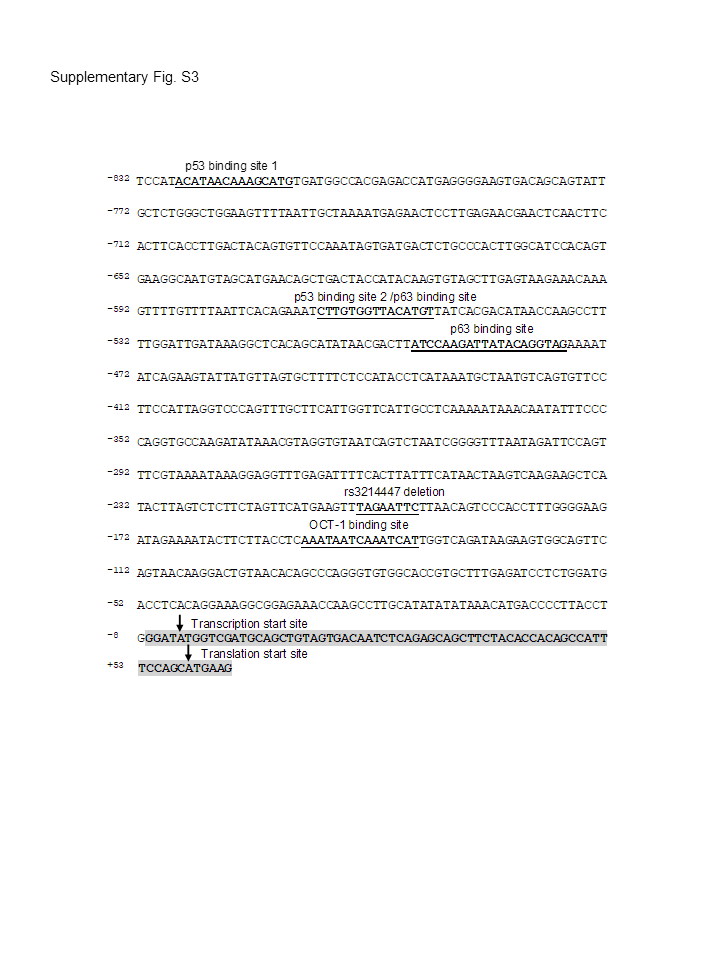

Supplement: Supplementary file 4 — Supplementary Figure 3 [file 41419_2020_2728_MOESM4_ESM.tif]

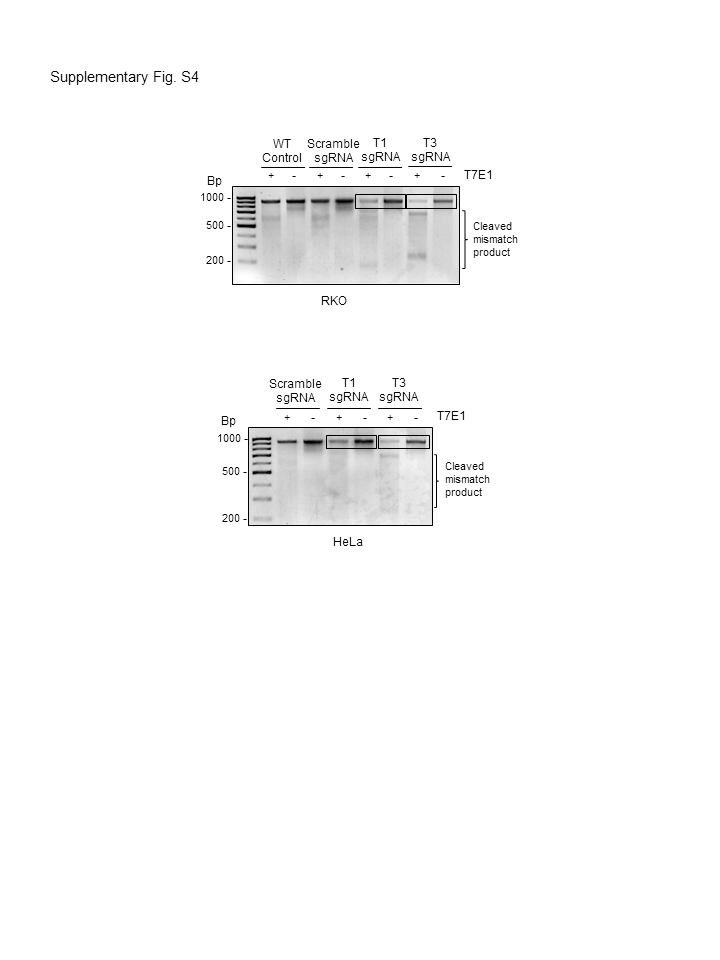

Supplement: Supplementary file 5 — Supplementary Figure 4 [file 41419_2020_2728_MOESM5_ESM.tif]
